# Chronic Low Dose Rate Ionizing Radiation Exposure Induces Premature Senescence in Human Fibroblasts that Correlates with Up Regulation of Proteins Involved in Protection against Oxidative Stress

**DOI:** 10.3390/proteomes2030341

**Published:** 2014-07-10

**Authors:** Olga Loseva, Emman Shubbar, Siamak Haghdoost, Bastiaan Evers, Thomas Helleday, Mats Harms-Ringdahl

**Affiliations:** 1 Division of Translational Medicine and Chemical Biology, Science for Life Laboratory, Department of Medical Biochemistry and Biophysics, Karolinska Institutet, Stockholm S-171 21, Sweden; E-Mails: olga.loseva@scilifelab.se (O.L.); thomas.helleday@scilifelab.se (T.H.); 2 Sahlgrenska Cancer Center, Department of Clinical Genetics, Institute of Biomedicine, Sahlgrenska Academy at University of Gothenburg, Gothenburg SE-41345, Sweden; E-Mail: emman.shubbar@gu.se; 3 Center for Radiation Protections Research, Department of Molecular Biosciences, The Wenner Gren Institute, Stockholm University, Stockholm S-106 91, Sweden; E-Mail: siamak.haghdoost@su.se; 4 Division of Molecular Carcinogenesis, The Netherlands Cancer Institute, Amsterdam 1066 CX, Netherlands; E-Mail: b.evers@nki.nl

**Keywords:** premature senescence, ionizing radiation, low dose chronic exposure, proteomics, human fibroblasts

## Abstract

The risks of non-cancerous diseases associated with exposure to low doses of radiation are at present not validated by epidemiological data, and pose a great challenge to the scientific community of radiation protection research. Here, we show that premature senescence is induced in human fibroblasts when exposed to chronic low dose rate (LDR) exposure (5 or 15 mGy/h) of gamma rays from a ^137^Cs source. Using a proteomic approach we determined differentially expressed proteins in cells after chronic LDR radiation compared to control cells. We identified numerous proteins involved in protection against oxidative stress, suggesting that these pathways protect against premature senescence. In order to further study the role of oxidative stress for radiation induced premature senescence, we also used human fibroblasts, isolated from a patient with a congenital deficiency in glutathione synthetase (GS). We found that these GS deficient cells entered premature senescence after a significantly shorter time of chronic LDR exposure as compared to the GS proficient cells. In conclusion, we show that chronic LDR exposure induces premature senescence in human fibroblasts, and propose that a stress induced increase in reactive oxygen species (ROS) is mechanistically involved.

## 1. Introduction

Radiation protection research is challenged by the demands to provide more precise risk estimates in the low dose and dose rate range for non-cancerous effects such as vascular diseases [1], cataracts [2] or reduced cognitive ability [3]. For doses in the mGy range, epidemiological studies will not be sensitive enough to reach these goals; however, when combined with a mechanistic understanding of the biological effects of low doses and dose rates, the limits of uncertainties of the present risk estimate may be better defined. The search for mechanisms behind low dose radiation induced non-cancerous adverse health effects may involve both the classical concepts of genotoxicity with expected stochastic dose response relation as well as mechanisms for bystander response, epigenetic effects and premature senescence. In this study, we hypothesize that low dose rates (≤15 mGy/h) will induce premature senescence in chronically exposed human fibroblasts.

Suzuki *et al*. have previously shown that acute high doses of ionizing radiation of human embryonic cells induces senescence like phenotypes and suggest that irreparable DNA damage triggers premature senescence [4].

In two recent studies, we have shown that chronic exposure to low dose rates induced premature senescence in human endothelial cells (HUVEC) [5,6]. A proteomic approach revealed that chronic radiation (4.1 mGy/h) induced DNA damage and oxidative stress that activated the p53/p21 pathway and inhibited the replicative potential [5] and when lower dose rates were used (1.4 and 2.1 mGy/h) inactivation of the PI3K/Akt/mTOR pathway was shown to accompany premature senescence [6], In parallel with the proteomic approach, a transcriptomic profiling was performed verifying that radiation induced oxidative stress and up regulation of GSH biosynthesis are characteristic for the first week of chronic exposure [7].

Cellular senescence has essential functions for organism ageing as well as for tumor control and substantial progress has been made in defining the mechanisms that triggers cells to enter senescence [8,9,10]. In the last few years, experimental data have been published suggesting that endogenous production of reactive oxygen species (ROS) contributes to senescence [11] and that oxidized nucleotides in the nucleotide pool are mechanistically linked to the induction of premature senescence [12]. These results support the view that the nucleotide pool is a critical target for ROS and unless the steady state levels of 8-oxo-dGTP were kept low through the action of the nucleotide pool sanitizing enzyme hMTH1, senescence was induced through signaling pathways resembling those for replicative senescence. We have recently shown that exposure to low doses and dose rates of low LET (linear energy transfer) radiation induced oxidative damage of the nucleotide pool (8-oxo-dGTP) in different cellular systems [13,14,15]. Doses in the mGy range induced a stress response that triggered endogenous ROS production and increased extra cellular levels of 8-oxo-dGTP. In parallel it was shown that the nucleotide sanitizing enzyme hMTH1 was induced.

The aims of this investigation were (a) to study the dose and dose rate dependence for induction of premature senescence (b) to further characterize the role of the endogenous oxidative stress response and (c) to analyze the subsequent changes in protein expression profile. Proliferating human fibroblasts were chronically exposed to low dose rates until they reached senescence. We used a proteomic approach to characterize differences at the molecular level between replicative senescence and radiation induced premature senescence. Our aim was to identify proteins that changed significantly in abundance, reflecting either synthesis or degradation in response to induction of senescence. The hypothesis that radiation induced oxidative stress causes premature senescence was further verified on glutathione synthetase (GS) deficient human fibroblasts, with the assumption that these cells are more sensitive to reactive oxygen species [16]. Here we show that low dose rate chronic exposure induces premature senescence in human fibroblasts. The proteomic analysis suggests that radiation induced premature senescence is closely related to replicative senescence. The increased sensitivity observed for the GS deficient cells supports the hypothesis that radiation induced endogenous formation of ROS induces premature senescence.

## 2. Experimental

### 2.1. Radiation Source

A cell culture incubator equipped with a custom-made ^137^Cs source was used for chronic exposure of the cells to 5 and 15 mGy/h. Cells designated as control were grown in the same incubator shielded by 15 cm of lead plates that reduced the dose rate to 0.005 mGy/h.

### 2.2. Cell Culture Condition and Cell Growth Kinetics

Normal human diploid foreskin fibroblast strain VH10 (Leiden University, Leiden, The Netherlands) was cultured in DMEM supplemented with 10% FBS, 100 U/mL penicillin and 0.1 mg/mL streptomycin (all media components from Invitrogen, Paisley, UK) at 37 °C under 5% CO_2_ atmosphere. To establish the growth rate kinetics, the cells were counted at regular intervals (every 7 days) until they became senescent. Linear regression analysis was used to test the linearity between the population doubling and the corresponding dose rate for each experiment. The significance of differences in dose responses was assessed by comparison of the curve slopes (Student’s *t*-test, *p* < 0.05). The growth rate kinetics for each dose rate was established using the equation: G = ln(n_1_/n_0_)/ln2, where n_0_ is the number of the cells seeded at day 0 and n_1_ is number of the cells that were counted at given days. Trypan blue exclusion assay was carried out to check cell viability. The experiment was started by inoculating 5 × 10^5^ cells at passage 12. These cells were exposed to chronic irradiation until they became senescent. Sham-treated control cells at 0.005 mGy/h dose rate were subjected to the same handling, both in and out of the incubator, as irradiated cells. Two independent set of experiments were performed: the first one only for analysis of growth kinetics (three separate repeats that started at passage 12) and the second (two separate repeats that started at passage 14) for growth kinetics, proteomics and detection of senescence markers.

The glutathione synthetase deficient fibroblast cell line (GS fibroblast) was isolated from a female patient with severe glutathione synthetase deficiency syndrome (indicated as patient 3 in [17]). The GS fibroblasts used in the study has 15% of the GS activity left as compared to the control cells (37.6 ± 14.8 pkatal/mg protein) The cell culture condition and treatment of the GS fibroblast was identical as for VH10 cells.

### 2.3. Senescence-Associated β-Galactosidase (SA-βgal) Assay

VH10 cells were washed twice in PBS, fixed at room temperature for 6–7 min in 2% formaldehyde/0.2% glutaraldehyde, then washed three times in PBS and incubated at 37 °C with SA-βgal staining solution (1 mg/mL 5-bromo-4-chloro-3-indolyl β-D-galactoside, Sigma-Aldrich, St Louis, MO, USA) in buffer containing 100 mM citric acid, 200 mM sodium phosphate, 5 mM potassium ferrocyanide, 5 mM potassium ferricyanide, 150 mM NaCl, and 2 mM MgCl_2_ at pH 6.0. Staining was evident after 4–6 h. The cells were washed with PBS and then with distilled water before microscope examination. The cells at passage 14 were used as a control.

### 2.4. Western Blot Analysis

VH10 cells were lysed in standard Laemmli buffer [18] supplemented by protease inhibitor cocktail tablet (Roche) and after centrifugation were subjected to Western blot analysis. Proteins were separated on Bis-Tris NuPAGE Novex 4%–12% gels (Invitrogen) in MES buffer at 150 V and then electrophoretically transferred to PVDF membranes using semi-dry transfer method. Blots were probed with the following antibodies (Santa Cruz Biotechnology, Santa Cruz, CA, USA): anti-p53 (mouse monoclonal, sc-126), anti-p21 (rabbit polyclonal, sc-397), anti-p16 (rabbit polyclonal, sc-468) and anti-actin (goat polyclonal, sc-1616). The blots were then incubated with horseradish peroxidase-conjugated secondary antibody (Thermo Scientific, Rockford, IL, USA) and protein bands were visualized using SuperSignal West Femto Maximum sensitivity substrate (Thermo Scientific). The chemiluminescence signal was registered with a CCD camera and image analysis was performed using Image gauge software.

### 2.5. Two-Dimensional Polyacrylamide Gel Electrophoresis (2DE)

The cells were lyzed in Mammalian lysis buffer (Quigen) supplemented with Benzonase nuclease, protease and phosphatase inhibitors and total protein fraction was precipitated in ice-cold acetone. Precipitated proteins were solubilized in solution composed of 8 M urea, 4% CHAPS, 4 mg/mL DTT, 1% IPG buffer 3–10 NL. Protein concentration was measured with Coomasie Plus protein assay kit (Pierce) and bovine serum albumin was used as standard.

100 µg protein was used for analytical gels, while 200 µg protein was used for the preparative gels. IEF was performed using 13 cm ready-made IPG strips with nonlinear pH 3–10 gradient and IPGphor focusing system (GE Healthcare Life-Sciences). The IPG strips were rehydrated for 12 h and IEF was performed for 30000 Volt hours. Before SDS-PAGE the IPG strips were first equilibrated for 15 min in 50 mM Tris-HCl pH 8.8, 6M urea, 30% glycerol, 2% SDS, 10 mg/mL DTT and then for 15 min in the same buffer with 25 mg/mL iodoicetamide instead of DTT. After equilibration the strips were placed on the top of vertical polyacrylamide 10% gels and embedded in 1% hot low-melt agarose in electrophoresis running buffer 25 mM Tris, 192 mM glycine, 0.1% SDS. SDS-PAGE was performed in Hoefer SE600 gel electrophoresis unit in 1 mm thick 16 × 14 cm gels at 20 mA per gel. Gels were stained using commercial Silver staining kit PlusOne and protocol from GE Healthcare Life-Sciences with modification according [19]. The samples were prepared from two independent experiments and two gels were run for each preparation.

Silver-stained gels were digitized using Luminescent Image analyzer LAS-1000plus (Fuji Film, Stamford, CT, USA). 2D gel imaging and analysis software PDQuest 8.0.1 (Bio-Rad, Hercules, CA, USA) was used for quantification of protein spots, gel to gel matching and identification of differences between the control and treated samples. Gel images were normalized so that the total density in gel images was made equal. Protein spots with changes greater than two-fold in magnitude compared to the control were excised from the gels and the proteins were identified by peptide mass fingerprinting.

### 2.6. MALDI-TOF Mass Spectrometry Analysis and Protein Identification

A silver staining method, which is compatible with MS, was used. This method omits the use of glutaraldehyde in the sensitization step and formaldehyde in silver impregnation step [19]. The stained protein spots were excised from silver preparative gels using stainless steel blades. A modified sample preparation method was used, which incorporates a destaining step to remove silver prior to in-gel digestion with trypsin [20]. Silver ions were removed with 100 µL 1:1 solution of 30 mM potassium ferricyanide and 100 mM sodium thiosulfate. The gel pieces were washed three times with water, then with 50% acetonitrile in 25 mM ammonium bicarbonate and dried on SpeedVac. Proteins were in-gel digested with sequencing grade-modified trypsin (12.5 ng/µL) (Promega, V511A) as described in-gel digestion protocol from Mass Spectrometry Facility of University of California at San-Francisco [21], except that reduction and alkylation steps were omitted because cysteines were carbamidomethylated on the equilibration step of 2-DE. After overnight incubation at 37 °C the resultant peptides were extracted with 50% acetonitrile/5% formic acid and dried in the vacuum centrifuge. The recovered peptides were purified and concentrated on C_18_ZipTips (Millipore) according to the manufacturer’s instructions. 

Mass spectra were recorded in positive reflection mode by using an Applied Biosystems MALDI-TOF Voyager-DE STR mass spectrometer equipped with a delayed ion extraction technology. α-Cyano-4-hydroxycinnamic acid was used as the matrix. The TOF was measured using the following parameters: 20 kV accelerating voltage, 200 ns delay, low mass gate 700 Da, and acquisition mass range 800–4000 Da. External calibration was performed using the Sequazyme Peptide Mass Standard kit with Angiotensin I (1,296.6853 Da) and ACTH clips 1–17 (2,093.0867 Da), 18–39 (2,465.1989 Da), 7–38 (3,657.9294 Da) and for internal calibration auto digestion peaks of bovine trypsin were used. The peptide mass profiles produced by MS were analyzed by using the programs Mascot [22], MS-Fit [23], and ProFound [24]. The monoisotopic peptide masses were compared with the theoretical masses derived from the NCBInr and SwissProt databases for human proteins. Search parameters included allowed mass accuracy 50 ppm, more than four peptide mass fits required for a protein match, consideration of one missed enzymatic cleavage, pI range of 3.0–10.0, and molecular mass range of 1–200 kDa. Accepted modification included carbamidomethylation of cysteine residues and methionine in oxidized form. Identification of proteins were based on MASCOT score and E-values, the observed pI and Mr (kDa) of the protein, the number of matching peptides and the total percentage of the amino acid sequence that those peptide covered. ID number and predicted protein function are found at UniProtKB [25]. Criteria used for protein identification followed the general guidelines for reporting proteomic experiments (MIAPE [26]). 

### 2.7. Heatmap and Hierarchical Clustering

Hierarchical clustering and heatmap visualization was performed using Matlab 2009b (Mathworks). The clustering was obtained by first calculating a Euclidian distance matrix of 50d, 65d and 75d sample levels of the 15 mGy/h experiment, normalized to their untreated controls. Next, linkage analysis was performed based on average distance.

## 3. Results and Discussion

### 3.1. Radiation-Induced Senescence in Normal Human Fibroblasts

**Figure 1:**
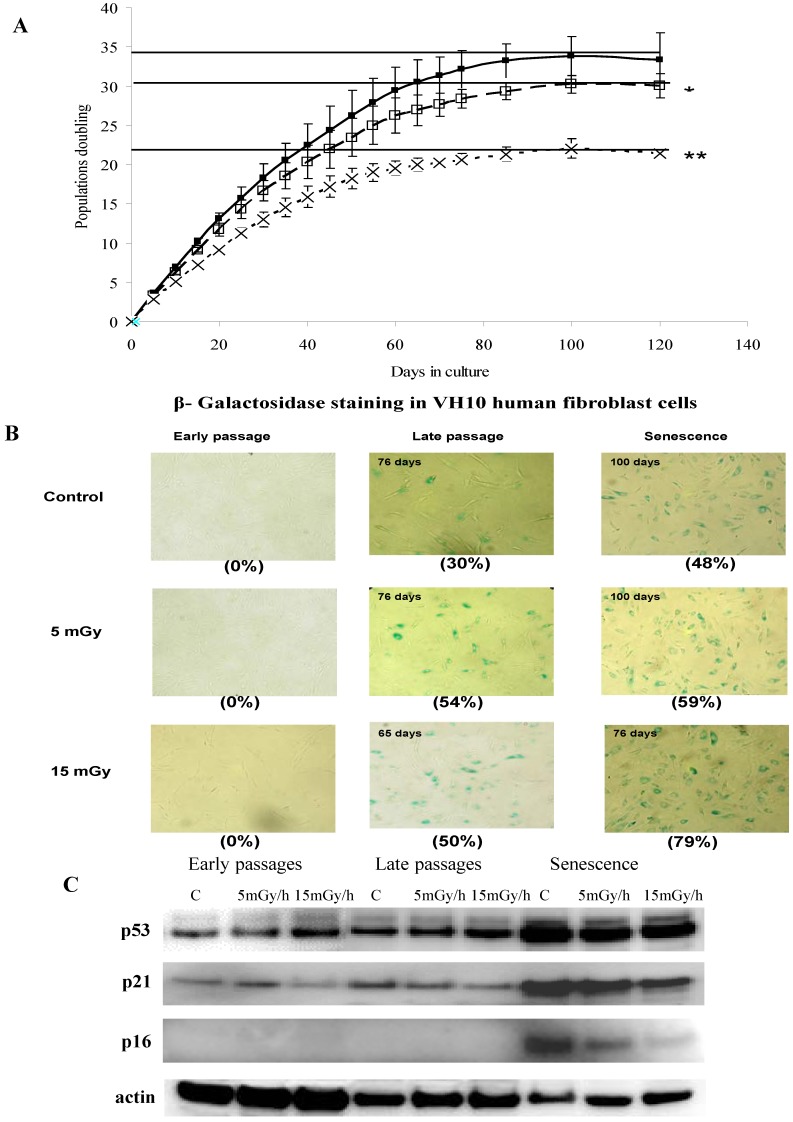
Panel (**A**): Growth rate kinetics of VH10 cells in response to chronic dose of 5 (□) and 15 (**×**) mGy/h γ-rays as well as for non-exposed cells (■). The results are based on 3 independent experiments (*n* = 3) for each dose rate that were started from passage 12. The slopes of growth rate for each experiment have been calculated and used to test the significance between the growth rates of non-exposed and exposed cells; Panel (**B**): The senescence-associated β-galactosidase staining of VH10 cells. Fibroblasts at early (20 days of culture), late and senescent passages (days of culture as shown in **C**) were subjected to *in situ* SA-βgal staining at pH 6 and examined by bright field microscopy. Cellular senescence is evident by flattened cell morphology, growth arrest and augmented senescence-associated β-galactosidase activity (numbers in brackets represent percent of β-galactosidase active cells); Panel (**C**): Western blots showing the expression of p53, p21 and p16. VH10 cells were harvested at early-, late-passages and senescent stages. Total protein extract were subjected to SDS-PAGE and Western blotting. The membranes were developed with antibodies for p53, p21, p16 and actin as control. Data are representative of two independent experiments.

**Figure A1:**
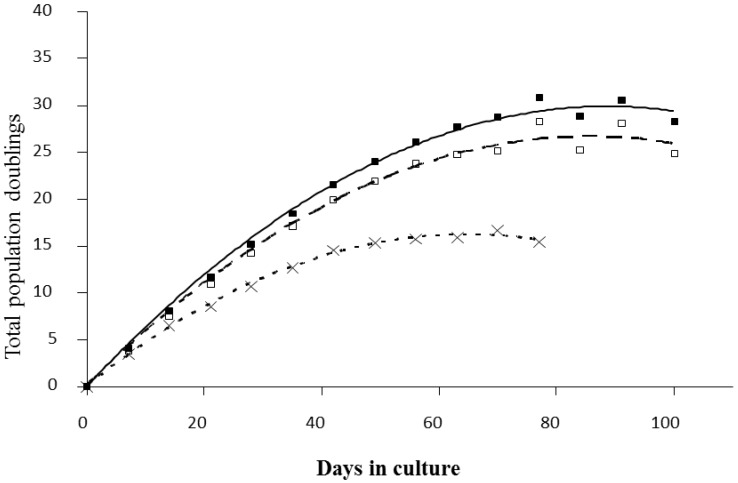
Growth rate kinetics of VH10 cells in response to chronic dose of 5 (□) and 15 (**×**) mGy/h γ-rays as well as for non-exposed cells (■). The results are based on 2 independent experiments for each dose rate that were started from passage 14. The exposed cells from this experiment were used for 2DE analysis and arrows indicated time points when the cells were taken for proteome analysis.

Cellular senescence is an irreversible cell-cycle arrest, activated in response to various types of stress [27]. Dysfunctional telomeres, oncogenic or stressful stimuli are known to trigger cellular senescence in normal human diploid cells during which they cease to proliferate and undergo a series of dramatic morphological, functional and gene expression changes [28]. The aim of this study was to investigate the effects of LDR chronic exposures on the proliferation of long term cultures of human fibroblasts and on the onset of senescence. To test this, we exposed proliferating human fibroblasts (VH10) chronically to either 5 mGy/h or 15 mGy/h. We found a dose and dose rate effect for the attenuation of the rate of proliferation in response to LDR chronic exposure and that the cells lost the ability to proliferate after fewer population doublings, compared to control cells (Figure 1A and Appendix Figure A1). In addition to irreversible growth arrest, senescent cells undergo distinctive changes in morphology and are characterized by positive staining for β-galactosidase activity at pH 6 [29]. We stained cells for senescence-associated β-galactosidase (SA-βgal) and found that the expression of SA-βgal correlated with the loss of proliferation and was observed after approximately 65 days of exposure to 15 mGy/h, while the corresponding values for cells exposed to 5 mGy/h or for control cells were 80 and 85 days of culture, respectively (Figure 1B).

The total accumulated dose corresponding to 15 mGy/h for 65 days or 5 mGy/h for 80 days was 23.4 or 9.6 Gy respectively. Thus, it may be concluded that for the cellular model system used here “high” doses and dose rates exceeding by far the normal background dose rates were needed to demonstrate induction of premature senescence. Radiation induced premature senescence has previously been shown for normal as well as cancer cells in response to acute exposures and high doses (for review see [30]).

In recent studies by Yentrapalli *et al*., where HUVEC cells were exposed under chronic conditions to low dose rates, premature senescence was induced at 4.1 mGy/h [5] as well as 2.4 mGy/h [6]. For the two comparable dose rates, 5 mGy/h for VH10 cells and 4.1 mGy/h for HUVEC, the latter were markedly more sensitive and entered senescence after a total dose of 6.2 Gy while the corresponding dose forVH10 cells was 7.8 Gy.

It is known that the expression of CDK inhibitors p16 (INK4a) is critical during replicative senescence [31] and p21 for high dose radiation-induced senescence [32] which is in turn regulated by p53 [33]. We analyzed expression levels of p53, p21 and p16 and found that expression of p53 and p21 increased in senescence cells compared to cells at late passages.

An increase in p53 and p21 was observed in HUVEC cells that entered radiation induced premature senescence [5].

The highest level of p16 was observed in cells undergoing replicative senescence and less so for cells undergoing LDR-induced senescence (Figure 1C), indicating that the radiation induced premature senescence may differ from replicative senescence in specific response pathways.

**Figure 2:**
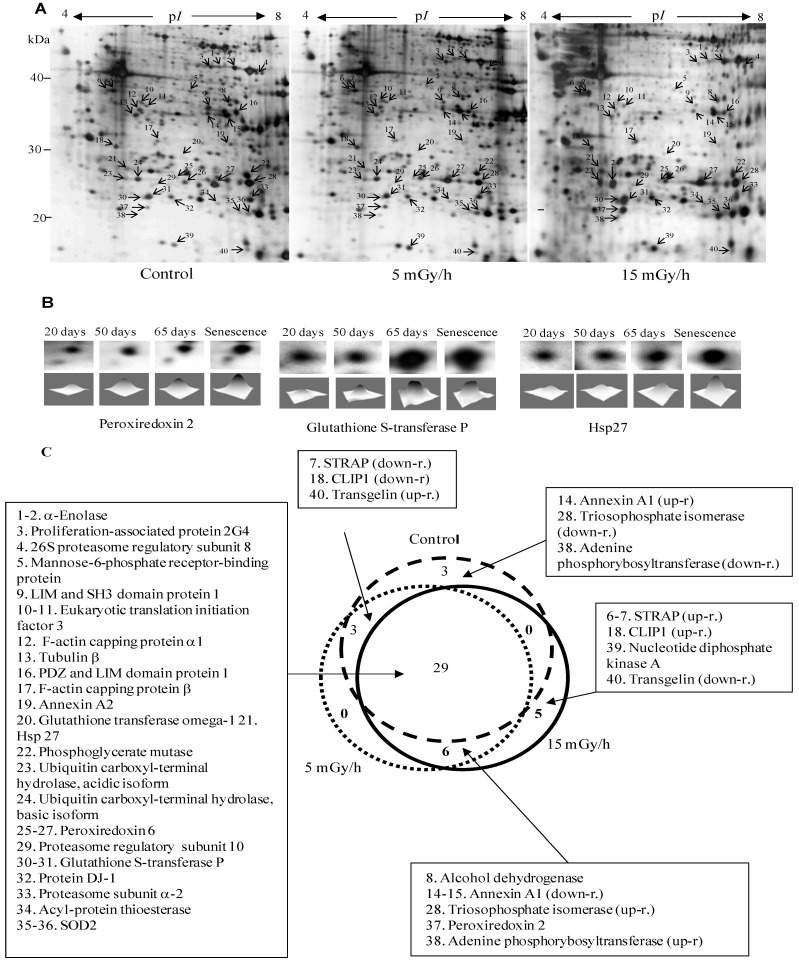
Panel (**A**): Representative 2D maps of proteins in control VH10 cells and after 65 days of low dose chronic exposure. 100 µg proteins were loaded and separated in the first dimension by IEF on IPG strips with nonlinear pH 3–10 gradient, then in the second dimension by SDS/PAGE on 10% polyacrylamide gels. Proteins were revealed by silver nitrate staining. The numbered proteins were identified by mass spectrometry and listed in Table 1; Panel (**B**): 2D gel images showing selected up-regulated proteins after 20, 50, 65 and 76 days (senescence) chronic exposure at 15 mGy/h; Panel (**C**): The diagram presents comparison of numbers of senescence-associated proteins in the control and two irradiated samples.

**Figure 3:**
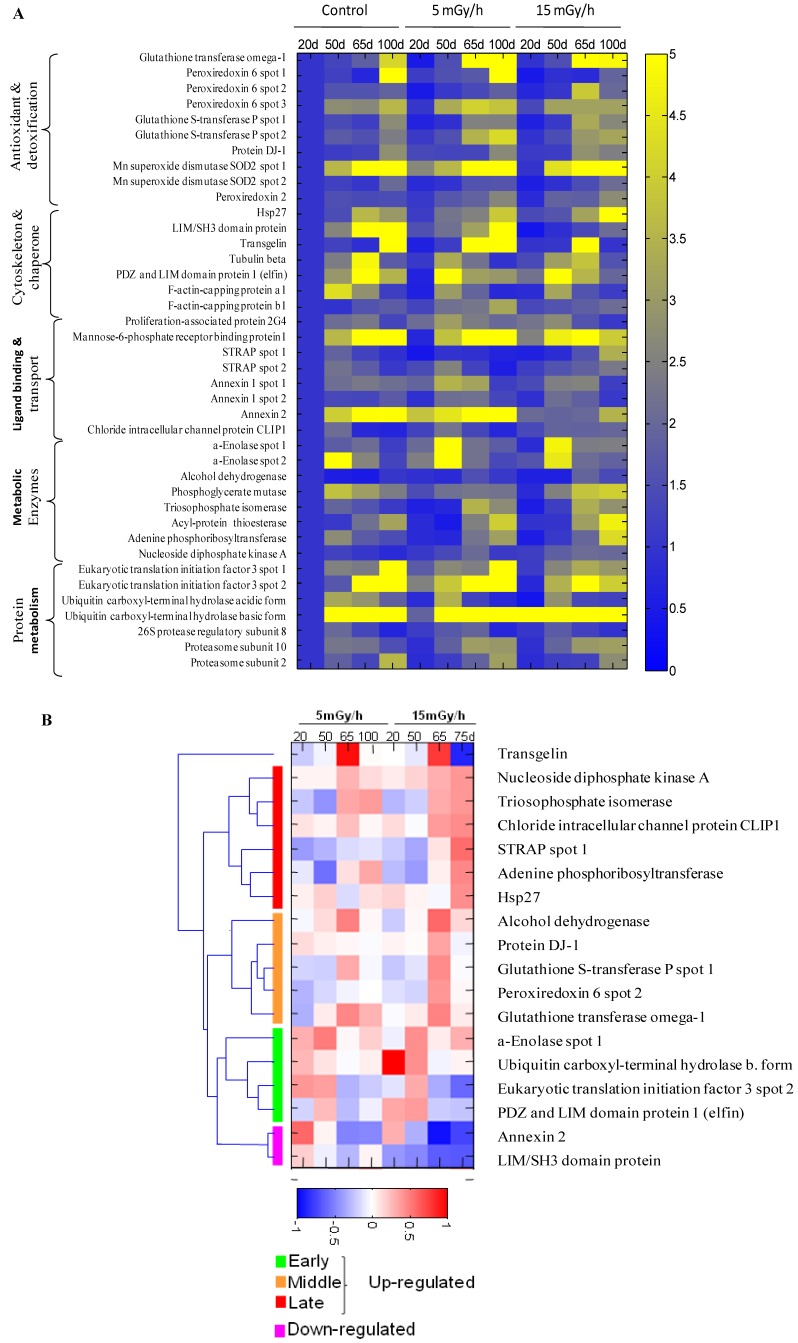
The heat map showing senescence-associated changes in relative level of differentially expressed proteins in the control and irradiated cells. Integrated intensity of protein spots on 2DE gels was determined by PDQuest 8.0.1 (Bio-Rad) at indicated time points for samples of control cells and cells continuously receiving 5 mGy/h or 15 mGy/h. Panel (**A**): The relative spot intensity of differentially regulated proteins in treated samples compared to untreated controls is shown. Proteins are grouped according to function; Panel (**B**): Unsupervised hierarchical clustering on the 15 mGy/h samples reveals groups of proteins that are up-regulated early, intermediate and late after irradiation. Depicted are log_10_ values of protein levels in treated cells normalized to levels in untreated cells of the same time point.

Induction of replicative and premature senescence in human fibroblasts was connected with changes in expression of other protein with functions in cell cycle control and proliferation. Proliferation associated protein 2G4, known also as Ebp1 (ErbB3 receptor-binding protein), belongs to DNA/RNA binding proteins and is implicated in cell growth, apoptosis and differentiation. The protein was down-regulated in all senescence cells (Figure 2 and Figure 3). Earlier it was shown that this protein disappeared in G0 arrested cells and that levels were restored after release from growth arrest [34]. Later it has been found that Ebp1-deficient mice displayed growth retardation [35]. The proliferation of fibroblast derived from knock out embryos was also decreased compare to wild type.

Thus, for both the control and chronically irradiated cells, the senescence-associated cell-cycle arrest correlated with the induction of p53 and the CDK inhibitors, p21 and p16 (INK4a), and down-regulation of Ebp1.

### 3.2. Effect of Ionizing Radiation on Fibroblast Proteome

To gain insights into the molecular mechanisms of LDR-induced senescence, we used a proteomic approach to determine which proteins were up- or down-regulated after LDR as compared to untreated control cells. Total protein fractions were isolated from control cells after 20, 50, 65 and 100 days of culture and from radiation exposed cells after 20, 50, 65, 100 days (5 mGy/h) and 20, 50, 65, 75 days (15 mGy/h) and analyzed by 2D gel electrophoresis (Figure 2A).

The gel image analysis was carried out using PDQuest software. This program allows automatic detection and quantification of protein spots as well as alignment and matching of gels. Differentially expressed proteins were identified by MALDI-TOF MS (Table 1). The majority of observed proteomic changes were similar between cells undergoing LDR induced premature senescence and control cells undergoing replicative senescence (Figure 2 and Figure 3A).

In order to identify alterations that were specific for the irradiated cells, we normalized the individual protein levels of the irradiated samples to the levels in the time point-matched control cells. Unsupervised clustering subsequently revealed distinct groups of proteins involved in early, intermediate and late irradiation specific responses (Figure 3B). Despite the evidence that replicative and stress-induced senescence are different phenotypes they share many cellular and molecular features [36] as can also be concluded from the proteomic data presented in this study.

The free radical theory of aging proposes that ROS cause damage to macromolecules, thus resulting in a decline of cellular functions and aging [37]. Many of the proteins identified to be up-regulated after LDR as well as in control cells during replicative senescence are proteins involved in the ROS response such as peroxiredoxins 2 and 6 (Prdx2 and Prdx6), the manganese-containing superoxide dismutase (MnSOD), glutathione S-transferase P (GST-P) and glutathione transferase omega (GST-O) (Figure 2 and Figure 3, Table 1). SOD enzymes catalyze the breakdown of superoxide into hydrogen peroxide and water and are therefore central regulators of ROS levels [38]. If genetic inactivation of MnSOD results in increased sensitivity of mutant mice to oxidative damage [39], over-expression of SOD in *S. cereviseae* and *Drosophila* can reduce oxidative damage and extend life span [40]. Peroxiredoxins play an important role in eliminating peroxides generated during metabolism or oxidative stress [41]. Prdx2 belongs to 2-cys Prdxs that use thioredoxin as a cofactor to reduce H_2_O_2_. It was shown that mice lacking 2-cys Prdx1 have a shortened lifespan as a result of severe hemolytic anemia and several malignant cancers [42]. Prx6, 1-cys enzyme that utilizes glutathione (GSH) to reduce H_2_O_2_ and organic hydroperoxides, was shown to protect epithelial cells from oxidative stress [43]. It is of interest to note that the proteomic analysis indicated that oxidative stress was also mechanistically involved in the premature senescence induced in the chronically exposed HUVEC cells [5].

Our analysis identified several proteins involved in the ROS response to be specifically important for the LDR-induced senescence, *i.e.*, DJ-1, Hsp27, GST-P, GST-O and some others (Figure 2 and Figure 3). Protein DJ-1, atypical peroxiredoxin-like peroxidase, scavenges oxidative stress by oxidizing itself to a more acidic form and/or by increasing glutathione synthesis through an increase of glutamate-cysteine ligase [44]. It was observed that over-expression of DJ-1 in animals or cultured cells prevent cell death, whereas knockdown or knock-out of DJ-1 increases the susceptibility to oxidative stress [45]. Hsp27 was significantly up-regulated in senescent cells after 15 mGy/h radiation (Figure 2 and Figure 3). An important function of Hsp27 is the ability to increase the resistance of cells to oxidative injuries by reducing lipid peroxidation, protein oxidation and F-actin architecture disruption [46,47]. The phenomenon depends on GSH and the up-regulation of glucose-6-phosphate dehydrogenase. The age-related accumulation of oxidized proteins is dependent on the balance between the accumulation of modified proteins and their elimination by protein degradation and repair system [48]. Ubiquitin carboxyl-terminal hydrolase isozyme (UCH-L1) is involved in processing and degradation of ubiquitinated proteins. UCH-L1 protein was presented in two isoforms with down-regulation of “acidic” and up-regulation of “basic” form in the process of aging of human fibroblasts (Figure 3).

**Table 1:** Identification of proteins differentially expressed after low dose chronic exposure in normal human fibroblast VH10 cells.

Spot Number	Protein Name	Protein ID	Theoretical pI	Theoretical Mr, kDa	Peptide Matches	Sequence Coverage %	Mascot Score	Protein Function
1	α-Enolase	P06733	7.0	47.2	15	36	120	Glycolysis
2	α-Enolase	P06733	7.0	47.2	20	33	197	Glycolysis
3	Proliferation-associated protein 2G4	Q9UQ80	6.1	43.8	14	27	66	Involved in cell cycle arrest/cell proliferation
4	26S protease regulatory subunit 8	P62195	7.1	45.6	12	36	85	Proteasome complex
5	Mannose-6-phosphate receptor-binding protein 1	O60664	5.8	28.1	15	48	141	Vesicle-mediated transport
6	Serine-threonine kinase receptor-associated protein	Q9Y3F4	5.0	38.4	9	41	70	mRNA processing, regulator of TGFβ pathway, cofactor of p53
7	Serine-threonine kinase receptor-associated protein	Q9Y3F4	5.0	38.4	8	32	65	mRNA processing, regulator of TGFβ pathway, cofactor of p53
8	Alcohol dehydrogenase	P14550	6.3	36.6	13	55	110	Glucose metabolic process
9	LIM and SH3 domain protein 1	Q14847	6.4	30.1	10	31	75	Actin-binding protein
10	Eukaryotic translation initiation factor 3	Q13347	5.4	36.5	9	30	71	Protein biosynthesis
11	Eukaryotic translation initiation factor 3	Q13347	5.4	36.5	10	32	73	Protein biosynthesis
12	F-actin-capping protein α-1	P52907	5.4	32.9	14	67	172	Regulation of cell motility
13	Tubulin beta	P07437	4.8	48.7	14	45	132	Cytoskeleton
14	Annexin A1	P04083	6.6	38.7	18	52	179	Regulation of apoptosis
15	Annexin A1	P04083	6.6	38.7	19	62	202	Regulation of apoptosis
16	PDZ and LIM domain protein 1	O00151	6.6	36.5	9	23	78	Cytoskeleton protein required for actin stress fiber formation
17	F-actin-capping protein subunit β	P47756	5.4	31.5	9	44	67	Actin-binding protein
18	Chloride intracellular channel protein CLIP1	O00299	5.1	27.4	16	70	183	Chloride ion channel, anti-apoptotic
19	Annexin A2	P07355	7.6	38.6	20	51	210	Stress response, regulation of apoptosis
20	Glutathione transferase omega-1	P78417	6.2	27.8	10	29	100	Metabolism of xenobiotics, antioxidant
21	Heat shock protein β-1 (Hsp27)	P04792	6.0	22.8	8	38	65	Involved in stress resistance and actin organization
22	Phosphoglycerate mutase	P18669	6.4	26.7	18	73	200	Glycolysis
23	Ubiquitin thiolesterase L1, acidic isoforms	P09936	5.3	24.8	14	72	131	Processing of ubiquitinated proteins; anti-apoptotic
24	Ubiquitin thiolesterase L1, basic isoforms	P09936	5.3	24.8	14	75	171	Processing of ubiquitinated proteins; anti-apoptotic
25	Peroxiredoxin 6	P30041	6.0	25.0	7	36	57	Antioxidant
26	Peroxiredoxin 6	P30041	6.0	25.0	13	64	144	Antioxidant
27	Peroxiredoxin 6	P30041	6.0	25.0	14	70	155	Antioxidant
28	Triosephosphate isomerase	P60174	6.4	26.7	15	58	177	Carbohydrate metabolism
29	26S proteasome subunit 10	O75832	5.4	20.4	8	49	58	Acts as a regulatory subunit of the 26S proteasome
30	Glutathione S-transferase P	P09211	5.4	23.6	9	48	94	Antioxidant, anti-apoptotic
31	Glutathione S-transferase P	P09211	5.4	23.6	10	56	146	Antioxidant, anti-apoptotic
32	Protein DJ-1	Q99497	6.3	19.9	8	43	60	Redox-sensitive chaperone and a sensor for oxidative stress
33	Proteasome subunit α type-2	P25787	6.9	26.0	8	44	70	Proteasome complex
34	Acyl-protein thioesterase 1	O75608	6.3	26.7	5	41	56	De-palmitoylation of signaling proteins
35	Superoxide dismutase Mn SOD2	P04179	8.3	24.7	7	36	69	Antioxidant, age-dependent response to ROS
36	Superoxide dismutase Mn SOD2	P04179	8.3	24.7	8	40	78	Antioxidant, age-dependent response to ROS
37	Peroxiredoxin-2	P32119	5.7	21.9	9	35	79	Antioxidant, anti-apoptotic
38	Adenine phosphoribosyltransferase	P07741	5.8	19.6	8	68	91	Nucleotide metabolism
39	Nucleoside diphosphate kinase A	P15531	5.8	17.3	8	61	90	Synthesis of nucleoside triphosphates other than ATP, tumor suppressor, cofactor of p53
40	Transgelin	Q01995	8.9	22.6	13	56	121	Actin-binding protein, senescence marker

The spot numbers refer to Figure 2A. Protein ID number and predicted protein function are found at UniProtKB [25]. The numbers of identified peptides matching predicted peptides and coverage of the entire protein sequence by the identified tryptic peptides are presented. Mascot Scores greater than 56 are considered significant (*p* ≤ 0.05).

Two proteins associated with p53, Ser-Thr kinase receptor-associated protein (STRAP) and tumor suppressor nucleoside diphosphate kinase (NDK) were up-regulated in the cells exposed at 15 mGy/h (Figure 2 and Figure 3). It was shown that NDK and its binding partner STRAP interact with p53 and potentiate p53 activity [49]. The p53 activation by NDK and STRAP is mediated by removing Mdm2, a negative regulator of p53.

It is well known that cellular senescence is accompanied with changes in the cytoskeleton structures and cell morphology. Transgelin, known also as SM22 protein, was identified in several screens for biomarkers of aging in mammalian cells. Transgelin cross-links actin filaments and stabilized actin in aging cells [50]. It is known that senescence cells have higher level of transgelin [51] and recent evidences suggest that this protein acts as tumor suppressor [52]. Two isoforms of transgelin were identified in 2-DE map of VH10 cells and one isoform was selectively regulated in radiated cells (Figure 3B). Chloride intracellular channel (CLIC) protein distantly related to omega-type glutathione-S-transferase [53] is also differentially regulated in senescent and premature senescent cell (Figure 3A,B).

### 3.3. Radiation Induced Stress Response

Recently, we have shown that doses in the mGy range induced stress response leading to oxidative damage of the nucleotide pool in three different cellular systems and that this damage could be coupled to an endogenous free radical production [13,14,15]. ROS induced damage on the nucleotide pool has recently been shown to induced senescence if not removed by the nucleotide pool sanitizing enzyme hMTH1 through pathways that were similar to those for replicative senescence [12].

Here, we show that chronic LDR induces premature senescence and hypothesize that stress induced production of ROS act as the aging effector. Support for this hypothesis is provided from the observed up-regulation of both glutathione transferases GST-P and GST-O in response to the LDR-induced endogenous stress response. Detoxification mechanisms, including the removal of electrophiles by glutathione transferase-catalyzed conjugation are protective mechanisms, assuring cell longevity [54]. Glutathione transferases play fundamental roles in the cellular detoxification of a wide range of exogenous and endogenous compounds. In addition to detoxification functions, some members of the GST super family can serve as peroxidases and isomerases [55]. GSH is a key component of an integrated antioxidant system that protects cells and tissues from oxidative damage [56,57]. GSH is a ubiquitous thiol-containing tripeptide and is synthesized in a two-step procedure, where the first step is catalyzed by glutamate-cysteine ligase and the second step is catalyzed by GS.

Based on the observation that several GSH dependent enzymes were up-regulated as the cells aged and entered senescence we hypothesized that cells with intrinsic low levels of GSH would enter radiation induced premature senescence after fewer population doublings compared to cells with normal levels of GSH. This hypothesis was tested on fibroblasts obtained from a subject with an inherited defect in glutathione synthetase [58]. Patients with severe GS deficiency suffer life threatening acidosis caused by the high production of 5-oxoproline and they are treated with vitamins C and E to boost their antioxidant levels, and bicarbonate to neutralize the acidosis [16,57,59].

**Figure 4:**
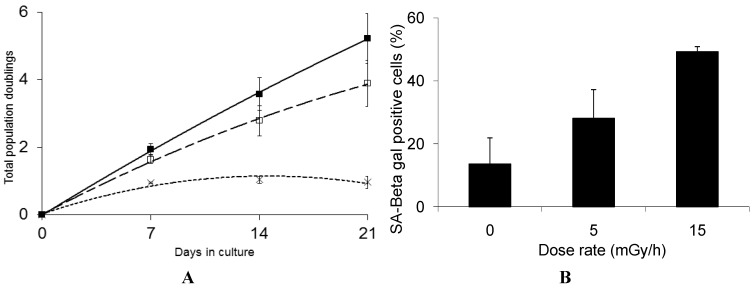
Panel (**A**): Growth rate kinetics of human fibroblast deficient in GS in response to chronic dose of 5 (□) and 15 (×) mGy/h γ-rays as well as for non-exposed cells (■). The results are based on 3 independent experiments (*n* = 3) for each dose rate; Panel (**B**): The quantification of SA-βgal staining in GS deficient fibroblast after 3 weeks of chronic exposure to γ-rays are based on 3 independent experiments (*n* = 3). The exposed cells were subjected to *in situ* SA-βgal staining at pH 6 and examined by bright field phase microscopy. The SA-βgal positive cells are presented as % of total number of the investigated cells.

Interestingly, we find that GS deficient cells are highly sensitive to low dose chronic exposure and rapidly lose the ability to proliferate when exposed to either 5 mGy/h or 15 mGy/h (Figure 4A).

The marked radiosensitivity reported here has not been observed in earlier studies on GS deficient cells, performed at high dose rates, where only moderate increased sensitivity was reported [60]. Radiation induced DNA damage and repair has also been studied in several GS deficient cell lines and the radiation effect on these endpoints is not significantly different from GS proficient cells [61,62]. For several of these cell lines, the total levels of low molecular weight free thiols are only moderately reduced as the GS deficiency causes elevated levels of cysteine and gamma-glutamyl cysteine, thus compensating low GSH content in terms of radical scavenging ability [63].

We suggest that the marked sensitivity to low dose rate chronic exposure observed for the GS deficient cells is caused by the reduced GSH levels causing suboptimal function of the GSH dependent enzymes such as GSH peroxidases and transferases involved in the protection against free radical induced damage [57]. Interestingly, the life span of *C. elegans* was significantly shortened when a group of five GSTs capable of catalyzing the lipid peroxidation product 4-hydroxynon-2-enal (4-HNE), were depleted using RNAi [64] and over-expression of GST was shown to extend the life span of *C. elegans* [65]. These results support the hypothesis that the marked sensitivity of the GS deficient cells is caused by their reduced defense against the increased oxidative stress induced by the LDR chronic exposure. The results presented in Figure 4B show that 50% of the GS deficient cells that stop proliferating in response to chronic LDR exposure at 15 mGy/h demonstrate senescence associated β-galactosidase staining. Compared to the VH10 cells, where 80% cells exposed to 15 mGy/h stained positive for β-galactosidase at the senescent stage, we cannot exclude that the rapid loss of proliferation of the GS deficient cells may in part be caused by alternative mechanism.

## 4. Conclusions

Although the analysis of the protein expression profiles obtained from replicative senescent cells compared to radiation induced premature senescent cells suggests that some unique characteristics correlate with the premature senescence, the overall response, as shown in Figure 2 and Figure 3 indicates that the pathways and regulation of radiation induced premature senescent phenotypes are closely related with the replicative senescent phenotype in cultures of human fibroblasts. The majority of proteins significantly differentially expressed are the same for the replicative senescent cells and the LDR-induced senescent cells. Hypothetically, this suggests that the chronic LDR exposures resulted in a stress response closely related to the replicative process of senescence observed for the control cells although at an increased speed. In favor of this is also the link, discussed above, between our observations of an endogenous stress response causing oxidative damage on the nucleotide pool and the rapid senescence in cells that have an impaired hMTH1 activity.

In summary, chronic exposure of human fibroblasts to low dose rates of ionizing radiation induced premature senescence as verified by loss of growth potential, and early induction of senescence-associated markers. A dose and dose rate effect was observed between the 5 and 15 mGy/h exposed cell cultures. The proteomic analysis indicated that the mechanism of radiation induced premature senescence was related to that of replicative normal senescence in this model system. The results support the hypothesis that radiation induced premature senescence was triggered by elevated levels of oxidative damage as a consequence of a stress response. There were however unique differences observed between the senescent and the premature senescent cells e.g., the p16 response and more studies are needed to reveal the mechanisms behind radiation induced premature senescence.
